# Drivers of understory species richness in reconstructed boreal ecosystems: a structural equation modeling analysis

**DOI:** 10.1038/s41598-020-68353-z

**Published:** 2020-07-14

**Authors:** Sanatan Das Gupta, Bradley D. Pinno

**Affiliations:** 1 Natural Resources Canada, Canadian Forest Service, Northern Forestry Centre, Edmonton, AB Canada; 2 Present Address: Department of Renewable Resources, University of Alberta, Edmonton, AB T6G 2H1 Canada

**Keywords:** Biodiversity, Forest ecology, Restoration ecology

## Abstract

Understory vegetation accounts for the most diverse part of the plant community in boreal forests and plays a critical role in stand dynamics and ecosystem functions. However, the ecological processes that drive understory species diversity are poorly understood and largely unexplored for reconstructed boreal ecosystems. The current study explored the relationships between understory species richness and biotic and abiotic factors in sites reclaimed after oil sands mining in northern Alberta, Canada, three and six growing seasons post-reclamation. Reclaimed sites with two main surface soils, forest floor mineral soil mix (FFMM) and peat mineral soil mix (PMM), were used along with post-fire benchmarks. A number of soil physicochemical (including nutrients) and vegetation properties were measured and considered in the a-priori hypothesis framework. Structural equation models (SEM) were used to evaluate the multivariate relationships. In general, the FFMM sites had greater species richness than the PMM sites, even six growing seasons after reclamation. A maximum 254% increase in graminoid and shrub cover was observed on FFMM between year 3 and 6 post-reclamation, whereas a maximum 137% increase in forb and bryophyte cover was recorded on PMM. The post-fire sites showed a significant increase (70%) only in shrub cover. Major driving factors of understory species richness varied among soil types. The SEM revealed a strong interdependency between species richness and soil and vegetation factors on FFMM with a positive control from soil N on species richness. In contrast, on PMM soil nutrients had a negative effect on species richness. Temporal changes in the drivers of species richness were mostly observed on FFMM through a negative vegetation control on species richness. The models and significant causal paths can be used in monitoring changes in understory species relationships in reclaimed sites and in identifying future research priorities in similar systems.

## Introduction

Understory vegetation is the most diverse part of the boreal forest plant community and contributes significantly to the biogeochemical cycles and plant community structure of forests^1–3^. Understanding the complex relationship among understory species richness, resource availability, and other abiotic and biotic factors is, therefore, crucial for biodiversity management in disturbance-prone ecosystems such as the boreal forest. Community dynamics in natural boreal ecosystems are largely fire-dependent with several stand-level factors such as species regeneration and recruitment, competition, moisture, light, and nutrient conditions altered in the post-fire ecosystem^4^. Changes in the abiotic conditions and forest floor heterogeneity, in particular, nutrient availability and organic matter quality, have been identified as the key factors controlling understory vegetation in post-fire stands; however, the drivers change as stands recover from disturbances^2^.

Effects of anthropogenic disturbance such as mining on understory vegetation differ significantly from fire disturbance. In the western boreal region of Canada, disturbances due to resource development, such as oil sands mining, have created a large-scale environmental footprint that is impacting forest regeneration and post-disturbance recovery. Oil sands mining requires removing both the aboveground and belowground material to access the ore. The subsequent reclamation processes involve reconstruction of the belowground system with upland and lowland-based soils salvaged during the mining process. Revegetation usually occurs in reclaimed sites through plantation (mostly conifers), seed in of deciduous trees, and regeneration of understory species either from seed in or from the transferred seed bank.

Propagule availability has been identified as the major source of species diversity in reclaimed sites^5^; however, soil properties such as bulk density (BD), texture, cation exchange capacity (CEC), C:N, and pH, and interactions with other species (competition and facilitation) have been shown to be important for the maintenance of species diversity on reclamation soils^6–9^. Recent studies showed that sites reclaimed with upland derived forest floor cover soils support greater understory species diversity, vegetation cover, and native species than other sites reclaimed with lowland derived peat cover soils^10–12^. Greater viable seed source, low C:N ratio, current and residual effects of coarse woody debris (CWD), and greater structural complexity were attributed to the diverse understory vegetation in forest floor cover soils^5, 10, 12, 13^. Lowland derived organic peat cover soils, on the other hand, have been found to support lower understory species richness and the differences were mainly attributed to the lack of viable upland seed banks, high organic carbon, and poor soil structure^5, 7, 9^.

Dispersal and recruitment mechanism contribute significantly to the development of local species pool in restored and newly reclaimed sites^14^. However, a number of recent studies have indicated that arrested succession may hinder and delay the development of targeted late-successional species in reclaimed sites^15^. Absence of meta-population and dispersal vectors, soil disturbance, dispersal barriers such as large infrastructure and roads, and synchronous biological events such as heavy seed rain, and species encroachment can all directly or indirectly influence plant community establishment in post-disturbed and newly reclaimed sites^16–19^. Seed dispersal has been shown to be more important than soil properties and resource availability during early stages colonization in mine reclamation^20, 21^. Seed bank and natural ingress studies conducted on reclaimed sites confirmed a considerably greater shrub richness on FFMM compared to PMM^22^. The lower species richness and very slow rate of increase in plant diversity on PMM may be related more to recruitment limitation than to dispersal limitation^23^ and this further necessitates the exploration of other means of enhancing local species pool such as direct plantation, seed and seedling enhancement, vegetative propagation, and animal dispersal on these sites.

Understory species response to soil nutrients also varies between FFMM and PMM. Errington and Pinno^10^ showed that fertilization increased total vegetation cover in both FFMM and PMM, but decreased species richness on FFMM and had no significant response on PMM. This suggests a negative effect of nutrients on species richness on FFMM. Since these soils are already favored by greater species diversity, at a higher level of nutrients, only a few nutrient-demanding species would outperform the generalists^24, 25^. The species richness-nutrient relationship on PMM would be neutral or positive due to less competition and more resources available for fewer species^10^.

Since the drivers of understory species richness are interdependent and function in a complex feedback loop, it is often difficult to identify the impacts from a linear or univariate relationship. Several previous reclamation studies tried to disentangle the complex relationships and found variable results indicating a resource dependency, substrate limitations, competition-facilitation, or bivariate associations between these factors^9, 10, 12, 26^. A multiple-effect hypothesis, however, has not been used to explain the mechanisms that regulate species richness and its temporal dynamics in the reclaimed systems. Structural Equation Modeling (SEM) has become very useful in identifying such a complex network of interdependent factors and is being used more frequently in ecological studies^27^. The key utility of SEM is its strength in confirming a-priori hypotheses and presenting the interrelationships in a graphical manner for better conceptualization of the modes and magnitudes of effects. SEM has also been used in reclamation studies in testing fundamental ecological theories and answering practical operational questions^26^. In the current study, we evaluated how the relationships between understory richness and different biotic and abiotic factors change in the two main reclamation soil types three and six growing seasons post-reclamation.

### A-priori hypothesis

**Figure 1:**
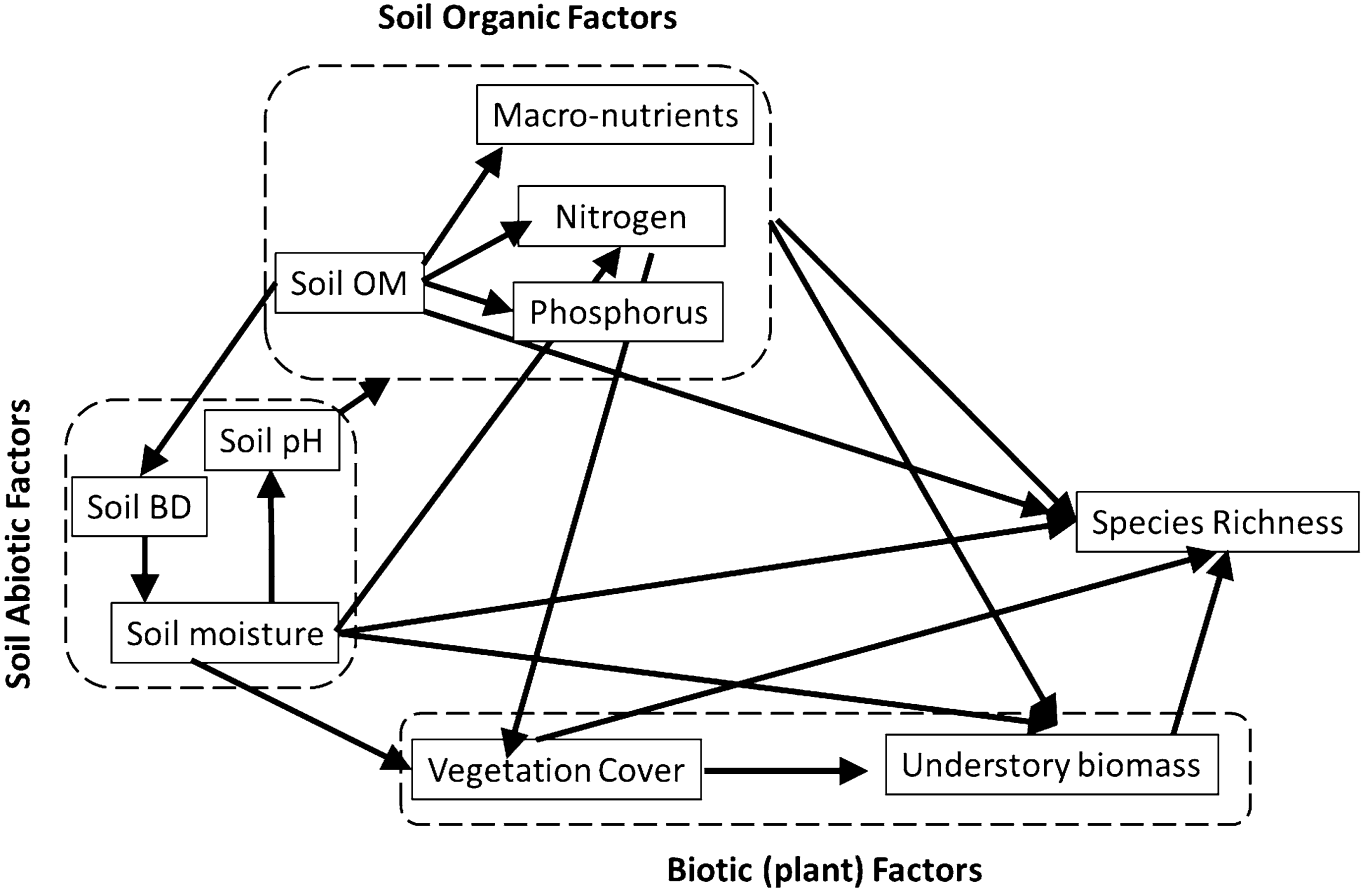
A-priori path models showing possible multivariate interrelationships between soil and plant factors that influence understory species richness in reclaimed and natural sites in northern Alberta.

In the *a-priori* model (Fig. 1) we hypothesized understory species richness is directly influenced by soil abiotic (bulk density, pH, and moisture), organic (organic matter and nutrients), and vegetation (cover and biomass) factors in different reclamation (FFMM and PMM) and natural soils. The mediation effects of these variables on species richness were also hypothesized through multiple indirect paths. We used the *a-priori* model to answer the following questions: (1) what abiotic and biotic factors drive understory species richness in reclaimed and natural post-fire sites? And (2) how do these relationships change with time since disturbance?

## Materials and methods

### Study area

We conducted this study on oil sands mining sites 75 km north of Fort McMurray, Alberta, Canada, and nearby natural forests. The reclamation site was an overburden dump with a total area of 88.6 ha constructed in 2011 using saline-sodic overburden materials removed during mining which was then capped by approximately 1.5 m of suitable sub-soil and then 0.5 m of selectively salvaged cover soils rich in organic matter. This cover soil was derived from nearby upland forests (Forest floor mineral soil mix, FFMM) or lowland peat bogs (Peat mineral soil mix, PMM). Barley (*Hordeum vulgare*) cover crop was aerially seeded in 2011 on all the soil types and white spruce were planted, deciduous trees (mainly trembling aspen) were allowed to seed in naturally as were understory plants.

Nearby (within 25 km) post-fire (Richardson Fire 2011) stands were used as reference sites to compare soil and vegetation dynamics with those in the reclaimed sites. The natural ecosystem is characterized as a boreal mixedwood forest with characteristic canopy species in the upland areas including pure or mixed stands of trembling aspen (*Populus tremuloides*) and white spruce (*Picea glauca*)^28^. The mean growing season temperature in this area is 16.8 °C and precipitation is 470 mm. The main soil type in natural upland sites is Grey Luvisol. The common (15% or more cover) understory vegetation in reclaimed sites were: native forbs such as *Achillea millefolium*, *Aster ciliolatus*, *Chamerion angustifolium*, *Equisetum arvense*, *Fragaria virginiana*, *Rubus pubescens, Rubus idaeus*; non-native forbs such as *Melilotus alba* and *Sonchus arvensis;* native grasses such as *Agropyron trachycaulum*, *Calamagrostis canadensis*, and *Carex siccata*; native woody shrubs such as *Salix bebbiana* and bryophytes such as *Ceratodon purpureus.*

In post-fire sites, the common species were: native forbs such as *Aralia nudicaulis, Chamerion angustifolium, Cornus canadensis*, *Linnaea borealis*, *Petasites palmatus* and *Rubus idaeus*; native shrubs such as *Viburnum edule*, *Alnus viridis* ssp. *crispa,* and *Rosa acicularis*, and bryophytes such as *Polytrichum juniperinum*. The post-fire sites had few to no non-native species.

### Sampling design

Sampling was done three growing seasons after reclamation, in 2013, and again in 2016 after six growing seasons. Sixteen 20 m × 20 m plots were established on each soil type at the reclaimed site. At each plot, measurements were done in four (4) 1 m × 1 m quadrats. Five post-fire stands were sampled in a similar manner in 2013. However, two fire plots were destroyed in a fire-break clearing in 2015 and six more plots were added in 2016. At each of the 1 m × 1 m quadrats, all vascular and non-vascular plants were identified to species level and vegetation cover was visually estimated to the nearest percent. Plant functional group and their native or non-native status species were also identified and recorded at each quadrat^10^. Species nomenclature including the description of growth forms and the designation of nativeness to Alberta follows Moss^29^. Biomass samples were collected using a 0.5 × 0.5 m clip plot close to the vegetation quadrats and later oven dried at 60 °C for seven days in the laboratory before taking the final weight measurements.

Soil samples from 0 to 10 cm were collected from all the quadrats. Soils were dried at 60 ± 5 °C and total carbon (TC), total nitrogen (TN), pH and electric conductivity (EC) were determined. TC and TN were measured using the dry combustion method by LECO CN analyzer. TC values were used as a proxy of organic matter (OM) in the statistical models^37^. Soil pH and EC were measured in 1:2 (1:5 for PMM) soil solution. Soil temperature and volumetric water content (VWC) were measured monthly using soil thermometer and TDR sensor (Field Scout 300 Soil Moisture Meter, Spectrum Technologies, USA).

Total inorganic nitrogen (TIN), phosphorus (P), potassium (K), sulfur (S), calcium (Ca), and magnesium (Mg) supply rates in soil were measured using the plant-root simulator (PRS) probes, Western Ag Innovations, Saskatoon, SK, Canada). One pair of cation and anion probes were installed in each quadrat and left buried for about 8 weeks (June–July 2014 and 2016). Upon retrieval, probes were cleaned with deionized water and sent back to the analytical laboratory in Saskatoon, Canada (Western Ag Innovations, SK, Canada). Inorganic NO_3_–N and NH_4_–N were determined using automated flow injection analysis. Other nutrients were analyzed using inductively-coupled plasma (ICP) spectrometry.

### Statistical analyses

Structural equation modeling (SEM) was used to assess the complex interrelationships between species richness and soil abiotic and organic properties, and understory vegetation properties in different reclamation soils and post-fire sites. SEM is a special format of the generalized linear model where multiple regression relationships are solved simultaneously to examine if a covariance matrix holds true based on a number of causal pathways set a priori. Understory species richness was used as the dependent variable, and the direct and indirect controls of soil and plant variables in different soil types were assessed through multi-group modeling. Vegetation cover and biomass include the sum of the contributions from all growth forms. All the biotic and abiotic factors and their plausible interactive pathways were considered in the initial model. A modified model was constructed by removing non-significant and less influential pathways when the hypothesized full model did not produce adequate fit. Temporal change in interactions was determined using the same model structure in different years. Standardized coefficients were used to compare the magnitude of effects in different years. Chi-square (χ^2^) test was used to assess the fit between predicted and observed covariance matrix. A χ^2^ with *P* > 0.05 indicates an acceptable model fit with the observed covariance structure. The fitted models were also assessed based on the Bentler’s comparative fit index (CFI) and Root mean squared error of approximation (RMSEA). A CFI > 0.95 and RMSEA < 0.10 were considered acceptable^27, 30^. The direct, indirect (mediation), and total (after accounting for both direct and indirect controls) effects of exogenous variables on species richness were also calculated. Since the sampling design of post-fire sites in 2013 was different and had fewer replications, SEM was developed only for the 2016 post-fire data. The SEM was implemented in AMOS 18.0 (Amos Development Corp.).

Mean differences in plant functional groups between soil types were analyzed using a one-way analysis of variance (ANOVA). Multiple group comparisons were performed using the Tukey post hoc test (α = 0.10). Patterns in soil nutrients were analyzed using principal component analysis (PCA). Multivariate differences in nutrients among soil types were analyzed using the multiresponse permutational procedure (MRPP) analysis. Bivariate correlation (Pearson’s r) between individual nutrients and ordination scores was also determined to show their interrelationships. Scores of the PCA axes (PCA1 and PCA2) explaining the highest variability were used as a proxy of the relative measure of soil nutrients in the SEM models. The ordination and MRPP analyses were conducted in PC-ORD software^31^, and the ANOVA and correlation analyses were conducted in R 3.2.3^32^.

## Results

### Understory vegetation

Understory species richness was greater on the FFMM than the PMM soils (Table 1). Post-fire sites had greater richness than PMM but did not differ with that on the FFMM sites. Forb cover was the highest among the functional groups in all the soil types. Species richness increased with time in both reclaimed and post-fire sites. Major temporal changes were detected in 2016 for forb and bryophyte cover on PMM (58% and 137% increase, respectively), graminoid and shrub cover on FFMM (153% and 254% increase, respectively) and forb cover in post-fire sites (20% increase) (Table 1). Greater variation (standard deviation) in vegetation and some edaphic factors was also observed in the FFMM sites compared to the PMM and post-fire sites (Tables 1 and 2).

**Table 1:** Species richness and vegetation cover (mean ± std. deviation) in the studied reclaimed and post-fire sites in northern Alberta in 2013 and 2016, 3 and 6 years post-disturbance, respectively.

	2013						2016					
	SR	Biomass (g m^−2^)	Forb (%)	Graminoid (%)	Shrub (%)	Bryophyte (%)	SR	Biomass (g m^−2^)	Forb (%)	Graminoid (%)	Shrub (%)	Bryophyte (%)
PMM	18.8^a^ (5.89)	30.5^a^ (23.5)	14.4^a^ (12.9)	0.86^a^ (1.33)	0.29^a^ (0.44)	5.18^a^ (6.26)	37.6^a^ (7.04)	26.6^a^ (12.3)	22.8^a^ (14.0)	3.79^a^ (3.54)	5.25^a^ (5.89)	12.3^a^ (7.48)
FFMM	28.5^b^ (7.20)	83.0^b^ (54.9)	45.8^b^ (18.9)	7.06^b^ (6.82)	2.42^a^ (3.23)	6.07^a^ (9.18)	45.7^b^ (5.62)	69.6^b^ (23.1)	39.5^b^ (15.3)	17.9^b^ (12.3)	8.57^ab^ (7.77)	9.08^a^ (11.0)
Post-fire	25.6^b^ (1.81)	49.0^a^ (16.8)	48.7^c^ (14.2)	3.83^a^ (3.96)	7.51b (2.26)	9.60^a^ (10.1)	40.4^a^ (4.57)	63.1^b^ (20.9)	38.0^b^ (9.51)	3.88^a^ (4.87)	12.7^b^ (4.77)	6.93^a^ (5.22)

Different letters indicate significant difference according to Tukey’s test (*P* < 0.10).

*SR* species richness.

**Table 2:** Soil properties (mean ± std. deviation) of the studied reclaimed and post-fire sites in northern Alberta in 2013 and 2016, 3 and 6 years post-disturbance, respectively.

	2013						2016					
	TC	TN	pH	N_av_	P_av_	K_av_	TC	TN	pH	N_av_	P_av_	K_av_
PMM	8.31^a^ (4.13)	0.27^a^ (0.15)	6.44^a^ (0.93)	4.10^a^ (1.39)	1.61^a^ (1.54)	20.8^a^ (15.0)	7.82^a^ (4.10)	0.29^a^ (0.21)	6.55^ab^ (0.98)	2.75^a^ (1.00)	0.95^a^ (1.22)	32.5^a^ (16.5)
FFMM	4.42^b^ (2.58)	0.19^a^ (0.09)	7.20^b^ (0.45)	4.34^a^ (1.59)	4.48^a^ (5.12)	30.1^a^ (18.1)	4.82^b^ (2.90)	0.23^a^ (0.14)	7.16^a^ (0.60)	4.22^b^ (1.67)	4.87^b^ (2.70)	93.9^a^ (124.5)
Post-fire	1.07^c^ (0.32)	0.07^b^ (0.01)	5.70^c^ (0.18)	4.30^a^ (2.19)	22.1^b^ (9.61)	305.5^b^ (153.1)	1.19^c^ (0.28)	0.07^b^ (0.01)	5.99^b^ (0.36)	3.48^ab^ (1.47)	12.5^c^ (6.63)	274.5^b^ (119.8)

Different letters indicate significant difference according to Tukey’s test (*P* < 0.10).

*N*_*av,*_* P*_*av,*_* and K*_*av*_ total inorganic N, P, and K, respectively as measured by the plant root simulator probes (PRS; µg 10 cm^−2^ 8 weeks^−1^); *TC* total carbon; *TN* total nitrogen.

**Figure 2:**
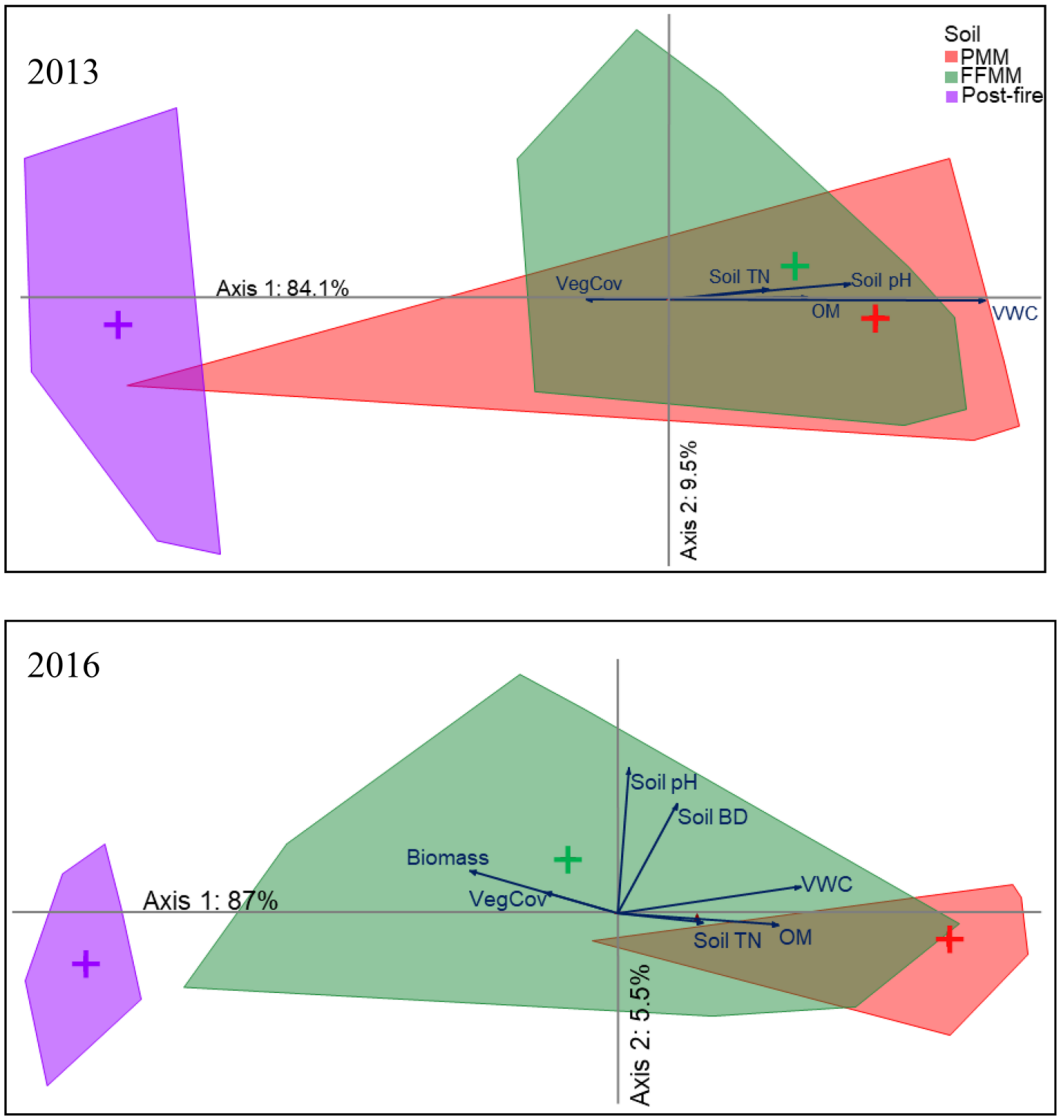
Principal component analysis showing the distribution of soil macronutrients as measured by PRS probes (µg 10 cm^−2^ 8 weeks^−1^) in oil sands reclaimed and post-fire sites in northern Alberta 3 and 6 years post-disturbance. *VegCov* total vegetation cover, *TN* total nitrogen, *BD* bulk density, *OM* organic matter, *VWC* soil moisture.

The FFMM sites had greater soil P and K than the PMM sites, but the post-fire sites had greater availability of these nutrients than both FFMM and PMM sites (Table 2). A decline in soil N, P, and K was observed between 2013 and 2016 in the PMM and post-fire sites, but not in the FFMM sites (Table 2). The PCA of soil nutrients produced ordination where axis 1 explained a maximum of 87% variation and axis 2 explained a maximum of 9.5% variation in nutrients (Fig. 2). The ordination scores of the PCA axis 1 mostly represented variations in soil P, K, S and base cations, and were used in further analyses (except the 2016 post-fire sites where Axis 2 was used) (Table S1).

Understory species richness on the FFMM sites was positively correlated with soil N but negatively correlated with OM, whereas on the PMM sites, species richness showed a negative correlation with soil N and moisture but a positive correlation with nutrient PCA1 (Table 3). Species richness was also negatively correlated with soil N and PCA1 but positively correlated with OM on the post-fire sites (Table 3).

**Table 3:** Bivariate relationship (Pearson’s r) between species richness, soil abiotic and organic properties, and understory plant properties in reclaimed and post-fire sites in northern Alberta.

	FFMM						
	Richness	OM	BD	VWC	N_av_	PCA1	Veg. cover
**Richness**							
Soil OM	**− 0.55**						
Soil BD	0.08	**− 0.92**					
Soil VWC	**− **0.28	**− **0.25	0.16				
N_av_	**0.55**	**− **0.29	0.13	**− **0.01			
PCA1	0.11	**− 0.56**	**0.42**	0.40	**− **0.26		
Veg. cover	0.16	0.16	0.12	0.15	0.14	**− **0.05	
Biomass	**− **0.18	**− **0.05	**− **0.04	**− **0.15	**0.39**	**− 0.55**	0.29
**PMM**							
Richness							
Soil OM	0.07						
Soil BD	0.01	**− 0.41**					
Soil VWC	**− 0.75**	**− **0.12	0.04				
N_av_	**− 0.56**	0.17	**− **0.26	**0.49**			
PCA1	**0.48**	0.06	0.13	**− 0.51**	**− 0.59**		
Veg. cover	**0.35**	0.13	**− **0.12	**− 0.51**	**− 0.51**	**0.31**	
Biomass	**− **0.01	0.01	**− **0.04	**− **0.18	0.20	**− **0.07	**0.50**
**Post-fire**							
Richness							
Soil OM	0.33						
Soil BD	**− **0.09	**− **0.33					
Soil VWC	**− **0.28	**− **0.15	**0**.44				
N_av_	**− 0.44**	**− 0.57**	0.49	0.30			
PCA1	**− 0.84**	**− **0.44	**− **0.05	0.41	**0.70**		
Veg. cover	**− 0.58**	**− **0.25	**− **0.13	0.29	**0.58**	**0.76**	
Biomass	**− **0.20	0.07	**0.73**	0.09	0.12	0.24	0.29

*OM* organic matter, *BD* bulk density, *VWC* soil moisture, *N*_*av*_ total inorganic N, *PCA1* ordination score of PCA axis 1.

### Structural equation models

A general co-variance structure of the a-priori model (Fig. 1) could not be met for all the sites (P < 0.05). However, retaining soil moisture and bulk density as abiotic factors, soil OM and nutrients as organic factors, and vegetation cover and biomass as biotic factors resulted in a significant model fit for all sites.

**Figure 3:**
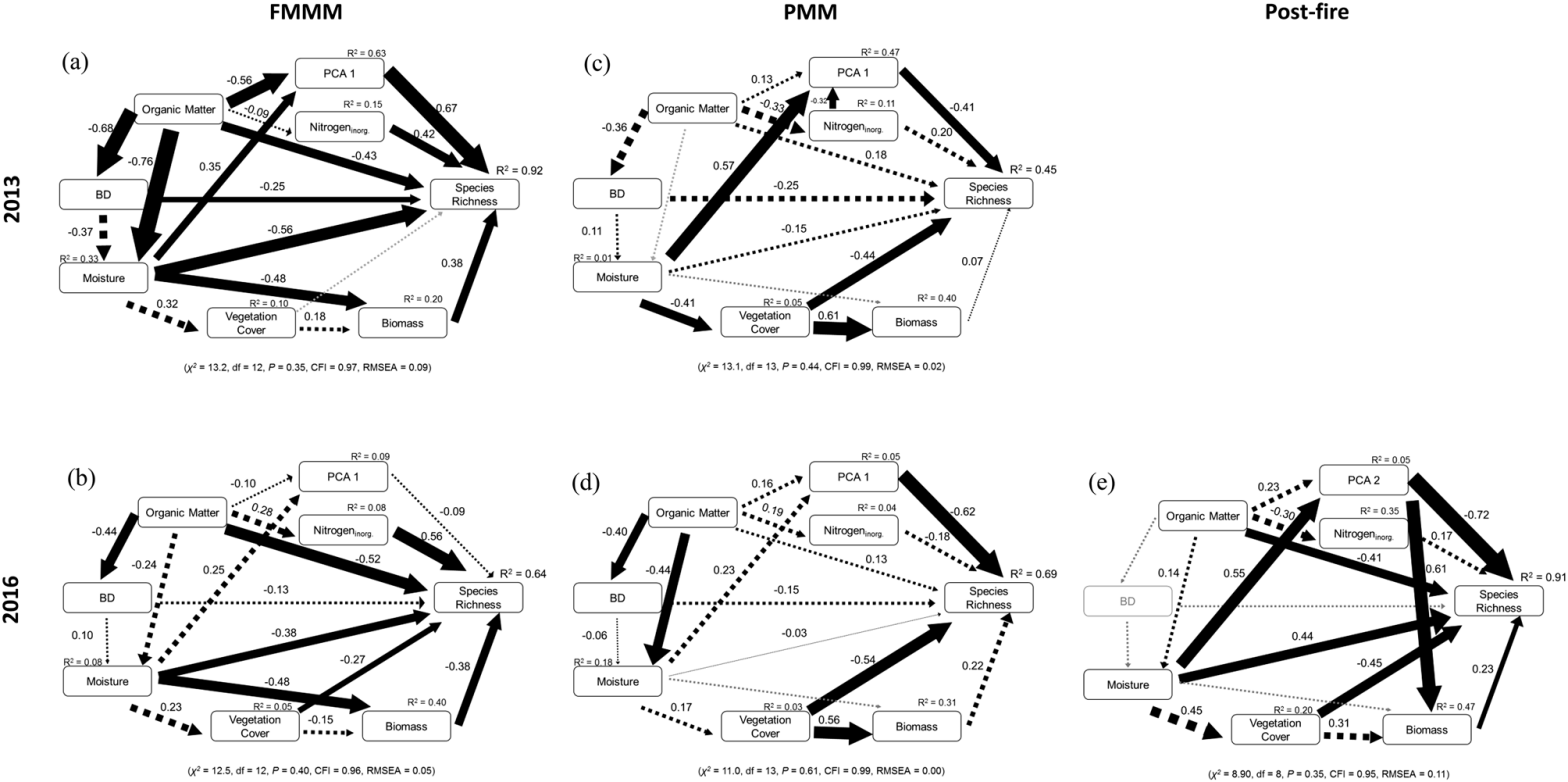
Path models (**a**–**e**) showing the effects of soil abiotic, organic, and plant biotic factors on understory species richness in reclaimed and post-fire sites 3 and 6 years post-disturbance. Solid lines indicate significant paths (*P* < 0.10) and broken lines indicate non-significant paths. No significant relationship was detected in post-fire sites in 2013. *PCA1* ordination score of PCA axis 1, *BD* bulk density.

Species richness on FFMM in 2013 showed a strong interdependency on all the abiotic, biotic, and organic factors (R^2^ = 0.92; Fig. 3a). Both soil N and nutrient PCA score (PCA1) and plant biomass had strong positive effects while OM, BD, and moisture had negative effects on species richness. A significant total effect on species richness was detected for OM, moisture, macronutrient and N availability, and understory plant biomass, whereas indirect effects of these variables on species richness were mostly non-significant (Table S1). The predictive ability of the 2016 model was weaker than the 2013 model (R^2^ = 0.64). Major changes in controllers of species richness in 2016 include a stronger positive effect of N, a weaker negative effect of soil moisture, negative effects of vegetation cover and understory plant biomass, and non-significant contribution of soil BD and PCA1 (Fig. 3b). Significant total effects were observed only for soil moisture and soil N (Table S2).

Species richness on PMM in 2013 was controlled by different factors than on FFMM. An overall R^2^ of 0.45 was achieved using the current model co-variance structure but with fewer significant paths (Fig. 3c). Soil nutrients (PCA1) and vegetation cover both had significant negative effects on species richness, whereas none of the other direct effects were significant (Table S3). Major changes in 2016 were observed through a stronger negative effect of soil nutrients (PCA1) and vegetation cover (Fig. 3d).

On post-fire sites, no significant relationship was detected between species richness and the biotic and abiotic factors in 2013 (data not shown). However, the SEM revealed significant interrelationships in 2016 through a negative control of OM, positive control of soil moisture, and understory biomass on species richness (Fig. 3e). A slight modification of the model structure was necessary for the 2016 model as the soil BD data was missing and modification indices suggested a path from macronutrient availability to understory biomass which was considered and may have theoretical merit in the studied system. Significant total effects on species richness were found for OM and soil moisture (Table S4).

## Discussion

Understory species richness showed variable responses to biotic and abiotic drivers and these responses differed by soil type. In FFMM and post-fire sites, the strongest effects on species richness were from OM and soil nutrients (including N), whereas on PMM, soil nutrients and vegetation cover were the strongest drivers of species richness. Species richness on FFMM showed a positive response to nutrient availability, especially N while this relationship was negative on PMM. The positive effect of soil N on species richness in the FFMM and post-fire sites corroborates studies conducted in forested ecosystems^33, 34^ and suggests that a similar mechanism of species diversity maintenance may be at work in the reclaimed sites. A positive relationship between N availability and the abundance of nitrophilic ruderal species in the post-disturbed (e.g., post-fire) sites is very common due to the large flush of nutrients. Total and inorganic N were also found elevated in the sites reclaimed with FFMM compared to the post-fire sites. The positive effect of nutrients on species richness on FFMM may also indicate an influence of heterogeneity-based niche differentiation in different understory species. The variable distribution of forest floor materials (woody debris and LFH layer) and viable seed propagules in the selectively salvaged FFMM soils are the key components of heterogeneity and may have contributed significantly to the observed relationship^7, 35^.

Very few direct relationships were detected for species richness on PMM suggesting that the resource dynamics in these organic soils are not tightly coupled with vegetation as was found on FFMM. Nitrogen and species richness were not linked in either year on PMM suggesting that understory richness may be related to other nutrients. Significant negative control of nutrient PCA axis (representing P, K, S, and base cations) on species richness independent of vegetation cover may indicate filtering effects on species pool. Similar negative control was reported by Gough and Grace^36^ in wetland ecosystems and by Laughlin, et al.^33^ in temperate pine ecosystem. Evidence from fertilized peat bogs showed that the effects of N and P availability on species richness are species-specific and often are not related to overall species richness^37^. McIntosh, et al.^38^ also did not find a significant link between nitrogen availability and understory plant composition in a boreal lodgepole pine-dominated forest and indicated the importance of other belowground drivers such as micronutrient availability and microbial functions. Pinno and Hawkes^11^ showed that the changes in soil P availability with stand age post-reclamation were related to total species richness and K availability was better related to non-native species richness. This further points to the importance of stoichiometric constraints on determining the effects of nutrient availability on species richness^39^.

Direct control of OM on species richness was negative on FFMM in both measurement years and did not have a significant effect on PMM. In general, soils with high organic matter should have greater nutrient supply and should support better plant growth. However, this may not be related to species richness if OM content is very high, as in the case of PMM (~ 4 to 12%), and does not have a direct relationship with nutrient availability. Germination strategy and life forms of plants have also been shown to be related to OM and mineral content of soils^2, 40, 41^. Mackenzie and Naeth^5^ found that greater mineral fraction and lower organic carbon in FFMM soils favor greater graminoid and ruderal species. A similar observation was also reported by Errington and Pinno^10^ where the OM and species richness relationship was negative on FFMM.

The effect of biomass (productivity) on species richness is positive in the FFMM and post-fire sites, although the reciprocal effect (the effect of species richness on biomass) was not significant. Understanding this interaction in young reclaimed sites is critical for biodiversity management. A spatial plant community study in the area indicated a possibility of facilitation among species on FFMM due to harsh environmental conditions compared to PMM where plants showed competition at similar spatial scale^42^. This might explain the observed non-significant effect of biomass on species richness on PMM. The positive control of biomass on species richness in post-fire sites corroborates findings from similar studies in boreal mixedwood ecosystems^43, 44^. Previous studies identified niche complementarity, facilitation, and selection effects are some of the main mechanisms responsible for the positive relationship between species richness, productivity, and ecosystem functions^44–47^. The combined effect of niche complementarity and facilitation is termed as niche complementarity effect and may operate simultaneously to bring changes in ecosystem functions^48^. Stressful post-disturbance conditions such as open canopy structure, less microtopographic heterogeneity, and high competition for resources (e.g., light, moisture, and nutrients) drive communities to share resources and consequently trigger positive complementarity through facilitation^49–53^. Conversely, less environmental stress (as in the case of PMM) would decrease facilitation as species become able to survive away from competing species and as a result would decrease the overall species diversity^54^. Reclamation study by Wang et al.^55^ reported a similar observation where they found greater plant biomass, soil nutrients, and microbial activities in plots with high species richness compared to the ones with low species richness and related this to stress-related plant facilitation. Indirect biotic facilitation has also been found in communities with high species diversity where competition among a specific set of species facilitated the survival and growth of other species^56, 57^.

The greater importance of organic factors such as soil nutrients and OM on species richness in FFMM and post-fire sites further indicate that understory species richness in these sites experiences a strong control from the legacy effects (i.e., past vegetation, seed propagule, and soil properties). These factors are also contributing to processes such as nutrient cycling, microbial activity, moisture availability, and spatial variability of abiotic resources^12^. Salvaged topsoils have been shown to be a good source of native seed propagules for reclamation purposes in many temperate, grassland, and alpine ecosystems^58–60^. The study by Mackenzie and Naeth^5^ in oil sands reclaimed sites showed almost 90% of the emergents on FFMM and 60% on PMM were from propagule banks which were found in the upper 10 cm of the cover soils. The same study also looked at species richness immediately after soil placement and found greater species richness on FFMM than PMM (29 species vs 16 species). A 10-year monitoring study on both soil types found significantly greater species richness on FFMM, whereas PMM had greater moss and lichen cover only^61^. A similar study by Anyia^62^ tested viable propagules in undisturbed natural soil and peat-based soils salvaged for reclamation and confirmed that seed bank alone would not be sufficient to reclaim PMM sites.

The weak association of species richness with OM, nutrients, or soil moisture on PMM (Figs. 3c,d; S2) suggests that local species pool development process is far behind than that on FFMM and natural sites^10^. When compared against a wider moisture gradient, pooled species richness (2013 and 2016 together) on PMM, however, showed a strong dependency on moisture (Table 3) which may indicate a high vulnerability of species diversity in these sites to climatic fluctuations (e.g. high temperature and drought conditions) unless a functional water and nutrient cycling mechanism is established^63^. The establishment of such functional linkage between vegetation and reconstructed soils may take as many as 25 years based on the recent reclamation studies^64–66^. The greater inter-annual variability in path mediation (i.e. inconsistency in path significance) on PMM compared to FFMM and natural soils could also be related to the high sensitivity of PMM to abiotic factors. Organic soils tend to have poor thermal conductivity and high thermal inertia that causes quick shifts between wet and dry conditions^67^. A recent study by Talon^68^ showed that temperature fluctuation on PMM is the greatest within the top 10–15 cm. The open canopy conditions (due to less vegetation cover) on PMM may even aggravate such local variabilities which can be detrimental for seed germination and further development of species pool on these sites.

The temporal changes in driving factors of species richness were observed mainly on FFMM through a stronger negative control from vegetation. This was expected as the changes in vegetation properties were most prominent on FFMM than on PMM. The causal paths linking species richness and other vegetation properties on PMM were similar in both measurement years indicating slower vegetation dynamics on PMM compared to FFMM and natural sites. Pinno and Hawkes^11^, however, reported an increase in species richness and forb cover on PMM until year 5 since reclamation. Given the lower relative changes in vegetation properties on PMM compared to FFMM, the effects of temporal changes in individual functional groups were probably masked by the inclusion of total cover and biomass in the SEM models. Further studies should consider analyzing these effects for individual functional groups, especially on PMM.

The findings from this study have several implications for plant biodiversity management on reclaimed sites. Since the understory vegetation community structure differed by reclamation soil types, future reclamation sites could include both reclamation soil types to maximize the diversity of habitats available. For example, sites reclaimed with PMM might generate an understory with higher bryophyte cover while sites reclaimed with FFMM have greater graminoid and forb cover. Pinno and Errington^23^ reported a greater establishment of trembling aspen (*Populus tremuloides*) on PMM compared to FFMM. A combination of FFMM and PMM sites may, therefore, create a balance in terms of creating a diverse understory in reclaimed sites. The lower understory species diversity and relatively slow temporal changes in species pool on PMM are concerning and should be addressed using assisted plant propagation and delivery techniques such as seed enhancement, seed packaging plugs and pucks, and planting large and nutrient-loaded seedlings^69^. Creating mosaic patches of propagule rich FFMM may also act as a seed source to less diverse PMM sites^10^.

The difference in soil nutrients and species richness relationships in the two studied reclamation soils can be used in developing soil-specific fertilization management guidelines to improve species diversity and control competitions in young reclaimed sites. Fertilization in organic matter rich soils (such as PMM) may not be beneficial for promoting overall understory species richness, at least during the initial stage of reclamation, and should be used cautiously for targeted species only. Physical properties of soils (e.g., bulk density and water holding capacity) should be used as a guiding template for controlling species richness when there is texturable mineral fraction (such as in FFMM). Deployment of operational use of reclamation soils may also consider this relationship between species richness and soil physical properties by carefully selecting slope and aspects where water availability during drier seasons would be important for plant survival and maintaining species diversity (e.g., low elevation and southern aspect).

Successful reclamation of disturbed landscapes depends very much on re-establishing plant-soil interactions and local species pools that are most suitable and have the greatest potential on specific sites and soil types^70^. The relationships observed in the natural post-fire sites helped to compare and justify the expected interactions in reclaimed sites at a similar temporal scale. Such comparisons can also be used to monitor changes in reclamation sites and to determine reclamation success at later successional stages.

The current study also indicated the use of conceptual causal models to infer understory community assembly mechanisms in heavily-disturbed ecosystems can help with the development of more reliable estimation of causal effect and may generate testable hypotheses on ecological restoration. Although the experimental design of this study was limited by site replication and unexpected changes in plot layout, especially in the post-fire sites, the findings from this research can be used to explain the short-term trend in understory vegetation changes in reclaimed sites and will guide any future research in this area.

## Supplementary information

Supplementary Information 1 (DOCX 1378 kb)

## References

[1] Nilsson M-C, Wardle DA (2005). Understory vegetation as a forest ecosystem driver: Evidence from the northern Swedish boreal forest. Front. Ecol. Environ..

[2] Hart SA, Chen HY (2006). Understory vegetation dynamics of North American boreal forests. Crit. Rev. Plant Sci..

[3] Gilliam FS (2007). The ecological significance of the herbaceous layer in temperate forest ecosystems. Bioscience.

[4] De Grandpré L, Gagnon D, Bergeron Y (1993). Changes in the understory of Canadian southern boreal forest after fire. J. Veg. Sci..

[5] Mackenzie DD, Naeth MA (2010). The role of the forest soil propagule bank in assisted natural recovery after oil sands mining. Restor. Ecol..

[6] Jung K, Duan M, House J, Chang SX (2014). Textural interfaces affected the distribution of roots, water, and nutrients in some reconstructed forest soils in the Athabasca oil sands region. Ecol. Eng..

[7] Forsch KBC (2014). Oil Sands Reclamation Using Woody Debris with LFH Mineral Soil Mix And Peat Mineral Soil Mix Cover Soils: Impacts on Select Soil and Vegetation Properties.

[8] MacKenzie M, Quideau S (2012). Laboratory-based nitrogen mineralization and biogeochemistry of two soils used in oil sands reclamation. Can. J. Soil Sci..

[9] Archibald, H. A. *Early ecosystem genesis using LFH and peat cover soils in Athabasca Oil Sands reclamation*. MSc Thesis, University of Alberta (2014).

[10] Errington RC, Pinno BD (2015). Early successional plant community dynamics on a reclaimed oil sands mine in comparison with natural boreal forest communities. Écoscience.

[11] Pinno B, Hawkes V (2015). Temporal trends of ecosystem development on different site types in reclaimed boreal forests. Forests.

[12] Chen HY, Biswas SR, Sobey TM, Brassard BW, Bartels SF (2018). Reclamation strategies for mined forest soils and overstorey drive understorey vegetation. J. Appl. Ecol..

[13] Pinno B, Das Gupta S (2018). Coarse woody debris as a land reclamation amendment at an oil sands mining operation in Boreal Alberta, Canada. Sustainability.

[14] Clark J (1999). Interpreting recruitment limitation in forests. Am. J. Bot..

[15] Boyes LJ, Gunton RM, Griffiths ME, Lawes MJ (2011). Causes of arrested succession in coastal dune forest. Plant Ecol..

[16] Holl KD (1999). Factors limiting tropical rain forest regeneration in abandoned pasture: Seed rain, seed germination, microclimate, and soil 1. Biotropica.

[17] Pajunen A, Virtanen R, Roininen H (2012). Browsing-mediated shrub canopy changes drive composition and species richness in forest-tundra ecosystems. Oikos.

[18] Hettenbergerova E, Hajek M, Zelený D, Jiroušková J, Mikulášková E (2013). Changes in species richness and species composition of vascular plants and bryophytes along a moisture gradient. Preslia.

[19] Walker LR, del Moral R (2009). Lessons from primary succession for restoration of severely damaged habitats. Appl. Veg. Sci..

[20] Lepš J, Michálek J, Rauch O, Uhlík P (2000). Early succession on plots with the upper soil horizon removed. J. Veg. Sci..

[21] Martineau Y, Saugier B (2007). A process-based model of old field succession linking ecosystem and community ecology. Ecol. Model..

[22] Naeth, M., Wilkinson, S., Mackenzie, D., Archibald, H. & Powter, C. Potential of LFH mineral soil mixes for reclamation of forested lands in Alberta. Oil Sands Research and Information Network, University of Alberta, School of Energy and the Environment, Edmonton, Alberta. OSRIN Report No. (TR-35, 2013).

[23] Pinno BD, Errington RC (2015). Maximizing natural trembling aspen seedling establishment on a reclaimed boreal oil sands site. Ecol. Restorat..

[24] Huston M (1980). Soil nutrients and tree species richness in Costa Rican forests. J. Biogeogr..

[25] Small CJ, McCarthy BC (2005). Relationship of understory diversity to soil nitrogen, topographic variation, and stand age in an eastern oak forest, USA. For. Ecol. Manage..

[26] Merlin M, Leishman F, Errington RC, Pinno BD, Landhäusser SM (2019). Exploring drivers and dynamics of early boreal forest recovery of heavily disturbed mine sites: A case study from a reconstructed landscape. New Forests.

[27] Grace JB, Anderson TM, Olff H, Scheiner SM (2010). On the specification of structural equation models for ecological systems. Ecol. Monogr..

[28] Beckingham, J. D. & Archibald, J. H. *Field Guide to Ecosites of Northern Alberta*. Vol. 5 (Natural Resources Canada, Canadian Forest Service, Northern Forestry Centre, Edmonton, Alberta, 1996).

[29] Moss, E. H. & Packer, J. G. *Flora of Alberta* 2nd edn. (University of Toronto Press, Toronto, Ontario, Canada, 1983).

[30] Shipley B (2016). Cause and Correlation in Biology: A User's Guide to Path Analysis, Structural Equations and Causal Inference with R.

[31] McCune, B. & Mefford, M. J. PC-ORD ver. 6.21, multivariate analysis of ecological data. *MjM Software, Gleneden Beach* (2011).

[32] R Development Core Team. *R: A language and environment for statistical computing* (R Foundation for Statistical Computing, Vienna, Austria, 2015). http://www.R-project.org/

[33] Laughlin DC, Abella SR, Covington WW, Grace JB (2007). Species richness and soil properties in *Pinus ponderosa* forests: A structural equation modeling analysis. J. Veg. Sci..

[34] Reich PB, Frelich LE, Voldseth RA, Bakken P, Adair EC (2012). Understorey diversity in southern boreal forests is regulated by productivity and its indirect impacts on resource availability and heterogeneity. J. Ecol..

[35] Kwak J-H, Chang SX, Naeth MA, Schaaf W (2015). Coarse woody debris increases microbial community functional diversity but not enzyme activities in reclaimed oil sands soils. PLoS ONE.

[36] Gough L, Grace JB (1998). Herbivore effects on plant species density at varying productivity levels. Ecology.

[37] Bedford BL, Walbridge MR, Aldous A (1999). Patterns in nutrient availability and plant diversity of temperate North American wetlands. Ecology.

[38] McIntosh AC, Macdonald SE, Quideau SA (2016). Understory plant community composition is associated with fine-scale above-and below-ground resource heterogeneity in mature lodgepole pine (Pinus contorta) Forests. PLoS ONE.

[39] Elser J (2000). Biological stoichiometry from genes to ecosystems. Ecol. Lett..

[40] Pugnaire FI, Lázaro R (2000). Seed bank and understorey species composition in a semi-arid environment: the effect of shrub age and rainfall. Ann. Bot..

[41] Nyland RD (2016). Silviculture: Concepts and Applications.

[42] Das Gupta S, Pinno BD (2018). Spatial patterns and competition in trees in early successional reclaimed and natural boreal forests. Acta Oecol..

[43] Reich PB (2001). Influence of logging, fire, and forest type on biodiversity and productivity in southern boreal forests. Ecology.

[44] Zhang Y, Chen HY, Taylor AR (2017). Positive species diversity and above-ground biomass relationships are ubiquitous across forest strata despite interference from overstorey trees. Funct. Ecol..

[45] Hooper DU (2005). Effects of biodiversity on ecosystem functioning: a consensus of current knowledge. Ecol. Monogr..

[46] Forrester DI (2014). The spatial and temporal dynamics of species interactions in mixed-species forests: From pattern to process. For. Ecol. Manage..

[47] Hector A, Bazeley-White E, Loreau M, Otway S, Schmid B (2002). Overyielding in grassland communities: Testing the sampling effect hypothesis with replicated biodiversity experiments. Ecol. Lett..

[48] Loreau M, Hector A (2001). Partitioning selection and complementarity in biodiversity experiments. Nature.

[49] Connell JH, Slatyer RO (1977). Mechanisms of succession in natural communities and their role in community stability and organization. Am. Nat..

[50] Callaway RM, Walker LR (1997). Competition and facilitation: A synthetic approach to interactions in plant communities. Ecology.

[51] Petchey OL, Gaston KJ (2006). Functional diversity: Back to basics and looking forward. Ecol. Lett..

[52] Gómez-Aparicio L (2004). Applying plant facilitation to forest restoration in Mediterranean ecosystems. Ecol. Appl..

[53] Wright AJ, Wardle DA, Callaway R, Gaxiola A (2017). The overlooked role of facilitation in biodiversity experiments. Trends Ecol. Evol..

[54] Michalet R (2006). Do biotic interactions shape both sides of the humped-back model of species richness in plant communities?. Ecol. Lett..

[55] Wang J (2014). Facilitation drives the positive effects of plant richness on trace metal removal in a biodiversity experiment. PLoS ONE.

[56] Aschehoug ET, Callaway RM (2015). Diversity increases indirect interactions, attenuates the intensity of competition, and promotes coexistence. Am. Nat..

[57] Lankau RA, Wheeler E, Bennett AE, Strauss SY (2011). Plant–soil feedbacks contribute to an intransitive competitive network that promotes both genetic and species diversity. J. Ecol..

[58] Ward S, Koch J (1996). Biomass and nutrient distribution in a 15.5 year old forest growing on a rehabilitated bauxite mine. Aust. J. Ecol..

[59] Scoles-Sciulla SJ, DeFalco LA (2009). Seed reserves diluted during surface soil reclamation in eastern Mojave Desert. Arid Land Res. Manag..

[60] Smyth C (1997). Early succession patterns with a native species seed mix on amended and unamended coal mine spoil in the Rocky Mountains of southeastern British Columbia, Canada. Arctic Alpine Res..

[61] Navus Environmental Inc (2009). LFH and Shallow Mineral Horizons as Inoculants on Reclaimed Areas to Improve Native Species Catch. Status Report Prepared for Syncrude Canada Ltd..

[62] Anyia, A. Final draft report: Germination and identification of indigenous plant species in Albian Sands Energy Inc. stripped soil used for reclamation of mined site. *Report prepared for Albian Sands Energy Inc., Fort McMurray, Alberta* (2005).

[63] Nenzén HK (2020). Projected climate change effects on Alberta's boreal forests imply future challenges for oil sands reclamation. Restor. Ecol..

[64] Rowland S, Prescott C, Grayston S, Quideau S, Bradfield G (2009). Recreating a functioning forest soil in reclaimed oil sands in northern Alberta: An approach for measuring success in ecological restoration. J. Environ. Qual..

[65] Sorenson P, Quideau S, MacKenzie M, Landhäusser S, Oh S (2011). Forest floor development and biochemical properties in reconstructed boreal forest soils. Appl. Soil. Ecol..

[66] Quideau S, Swallow M, Prescott C, Grayston S, Oh S-W (2013). Comparing soil biogeochemical processes in novel and natural boreal forest ecosystems. Biogeosciences.

[67] Eggelsmann R (1973). The thermal constant of different high-bogs and sandy soils. Proc. 4th Int. Peat Congr..

[68] Tallon L (2014). Spatial Variability of Thermal Properties in Reclamation Cover Systems.

[69] Schoonmaker, A. *et al.* Alternative Native Boreal Seed and Plant Delivery Systems for Oil Sands Reclamation. Oil Sands Research and Information Network, University of Alberta, School of Energy and the Environment, Edmonton, Alberta. OSRIN Report No. TR-55, pp 61. https://hdl.handle.net/10402/era.40099. (2014). Accessed 19 Mar 2019.

[70] Pinno BD, Li EH, Khadka B, Schoonmaker A (2017). Germination and early growth of boreal understory plants on 3 reclamation soil types under simulated drought conditions. Native Plants J..

