# Supplementary material for: Drivers of understory species richness in reconstructed boreal ecosystems: a structural equation modeling analysis
Source: Sci Rep. 2020 Jul 14;10:11555. doi: 10.1038/s41598-020-68353-z (PMC7360749; doi:10.1038/s41598-020-68353-z)
Supplement: Supplementary file 1 — Supplementary Information 1 (DOCX 1378 kb) [file 41598_2020_68353_MOESM1_ESM.docx]

**Supplementary material**

**Drivers of understory species richness in reconstructed boreal ecosystems: A structural equation modeling analysis**

Sanatan Das Gupta and Bradley D. Pinno

Table S1: Pearson’s correlation coefficients for PCA axes scores versus soil nutrients. Significant correlations are shown in bold.

|  | 2013 | | | | | | 2016 | | | | | |
| --- | --- | --- | --- | --- | --- | --- | --- | --- | --- | --- | --- | --- |
|  | FFMM | | PMM | | Post-fire | | FFMM | | PMM | | Post-fire | |
|  | Axis1 | Axis2 | Axis1 | Axis2 | Axis1 | Axis2 | Axis1 | Axis2 | Axis1 | Axis2 | Axis1 | Axis2 |
| Total inorganic N | -0.16 | 0.22 | -0.41 | 0.12 | 0.67 | 0.07 | -0.39 | **0.62** | -0.31 | 0.24 | 0.31 | 0.33 |
| Phosphorus (PO_4_^-^) | **-0.82** | **0.95** | **-0.63** | **0.95** | -0.68 | **0.95** | **-0.65** | -0.07 | **-0.65** | -0.22 | -0.41 | **-0.80** |
| Potassium (K^+^) | **-0.83** | **0.53** | **-0.75** | 0.02 | **-0.94** | 0.48 | **-0.64** | **-0.43** | **-0.79** | **-0.61** | -0.50 | **-0.73** |
| Sulfur (SO_4_^2-^) | **0.90** | -0.32 | 0.20 | 0.32 | **-0.95** | 0.54 | **0.94** | **-0.48** | **0.68** | **-0.49** | 0.15 | **-0.88** |
| Calcium (Ca^2+^) | 0.03 | 0.13 | **-0.44** | 0.22 | 0.11 | 0.64 | **0.59** | 0.20 | -0.18 | 0.01 | 0.22 | 0.08 |
| Magnesium (Mg^2+^) | -0.06 | 0.19 | 0.05 | 0.07 | 0.55 | 0.44 | **0.65** | -0.12 | 0.22 | 0.22 | 0.06 | 0.16 |

Table S2: Direct, total and indirect effects of soil and plant factors on species richness in the sites reclaimed with FFMM. Both significant and non-significant paths are shown. * = P ≤ 0.10; ** = P ≤ 0.05; *ꜞ = P ≤ 0.005; NA = not applicable

| Effects | Estimates | | SE | | Std. Effect | |
| --- | --- | --- | --- | --- | --- | --- |
|  | 2013 | 2016 | 2013 | 2016 | 2013 | 2016 |
| Soil OM → Species Richness |  |  |  |  |  |  |
| Total | -1.34** | -0.41 | 0.58 | 1.09 | -0.39 | -0.21 |
| Direct | -1.46*ꜞ | -1.03*ꜞ | 0.52 | 0.36 | -0.42 | -0.51 |
| Indirect | 0.12 | 0.61 | 1.37 | 1.85 | 0.03 | 0.31 |
| Soil BD → Species Richness |  |  |  |  |  |  |
| Total | -1.13 | -3.22 | 8.48 | 16.23 | -0.03 | -0.17 |
| Direct | -7.13** | -2.44 | 3.32 | 3.33 | -0.24 | -0.13 |
| Indirect | 6.01 | -0.78 | 5.69 | 3.51 | 0.21 | -0.04 |
| Soil Moisture → Species Richness |  |  |  |  |  |  |
| Total | -0.61** | -0.37** | 0.25 | 0.30 | -0.55 | -0.39 |
| Direct | -0.62*ꜞ | -0.36** | 0.12 | 0.16 | -0.56 | -0.38 |
| Indirect | 0.01 | -0.01 | 0.22 | 0.28 | 0.01 | -0.01 |
| Soil N → Species Richness |  |  |  |  |  |  |
| Total | 2.30** | 7.85** | 1.36 | 7.85 | 0.42 | 0.56 |
| Direct | 2.30*ꜞ | 7.85*ꜞ | 0.47 | 2.26 | 0.42 | 0.56 |
| Indirect | NA |  | NA |  | NA |  |
| Nutrient PCA1 → Species Richness |  |  |  |  |  |  |
| Total | 6.09** | 0.67 | 2.73 | 3.51 | 0.66 | 0.14 |
| Direct | 6.09*ꜞ | -0.43 | 1.21 | 0.96 | 0.66 | -0.09 |
| Indirect | NA | 1.11 | NA | 2.17 | NA | 0.23 |
| Vegetation cover → Species Richness |  |  |  |  |  |  |
| Total | 0.01 | -0.09 | 0.17 | 0.24 | 0.01 | -0.21 |
| Direct | -0.04 | -0.11* | 0.03 | 0.06 | -0.10 | -0.27 |
| Indirect | 0.04 | 0.02 | 0.19 | 0.05 | 0.10 | 0.06 |
| Plant biomass → Species Richness |  |  |  |  |  |  |
| Total | 0.04* | -0.09 | 0.02 | 0.14 | 0.54 | -0.38 |
| Direct | 0.03*ꜞ | -0.09* | 0.01 | 0.04 | 0.37 | -0.38 |
| Indirect | 0.01 | NA | 0.02 | NA | 0.17 | NA |

Table S3: Direct, total and indirect effects of soil and plant factors on species richness in the sites reclaimed with PMM. Both significant and non-significant paths are shown. * = P ≤ 0.10; ** = P ≤ 0.05; *ꜞ = P ≤ 0.005; NA = not applicable

| Effects | Estimates | | SE | | Std. Effects | |
| --- | --- | --- | --- | --- | --- | --- |
|  | 2013 | 2016 | 2013 | 2016 | 2013 | 2016 |
| Soil OM → Species Richness |  |  |  |  |  |  |
| Total | 0.18 | 0.31 | 1.33 | 0.62 | 0.11 | 0.16 |
| Direct | 0.27 | 0.21 | 0.36 | 0.31 | 0.17 | 0.11 |
| Indirect | -0.09 | 0.08 | 2.28 | 0.83 | -0.06 | 0.04 |
| Soil BD → Species Richness |  |  |  |  |  |  |
| Total | -4.43 | -1.81 | 21.8 | 6.75 | -0.27 | -0.11 |
| Direct | -4.04 | -1.92 | 3.54 | 2.65 | -0.25 | -0.11 |
| Indirect | -0.39 | 0.11 | 15.8 | 2.36 | -0.02 | 0.00 |
| Soil Moisture → Species Richness |  |  |  |  |  |  |
| Total | 0.11 | -0.13 | 0.70 | 0.69 | 0.22 | -0.10 |
| Direct | -0.07 | -0.04 | 0.13 | 0.21 | -0.15 | -0.03 |
| Indirect | 0.03 | -0.09 | 0.61 | 0.29 | 0.07 | -0.07 |
| Soil N → Species Richness |  |  |  |  |  |  |
| Total | 1.66 | 1.27 | 4.65 | 3.74 | 0.32 | 0.17 |
| Direct | 1.00 | -1.27 | 1.19 | 1.05 | 0.19 | -0.17 |
| Indirect | 0.65** | NA | 3.49 | NA | 0.13 |  |
| Nutrient PCA1 → Species Richness |  |  |  |  |  |  |
| Total | -4.96* | -7.98* | 21.1 | 11.2 | -0.41 | -0.64 |
| Direct | -4.96* | -7.98*ꜞ | 3.44 | 1.75 | -0.41 | -0.64 |
| Indirect | NA | NA |  | NA |  |  |
| Vegetation cover → Species Richness |  |  |  |  |  |  |
| Total | -0.16 | -0.15* | 0.52 | 0.20 | -0.39 | -0.39 |
| Direct | -0.17* | -0.21*ꜞ | 0.11 | 0.06 | -0.43 | -0.53 |
| Indirect | 0.01 | 0.05 | 0.44 | 0.21 | 0.04 | 0.13 |
| Plant biomass → Species Richness |  |  |  |  |  |  |
| Total | 0.01 | 0.15 | 0.37 | 0.45 | 0.06 | 0.24 |
| Direct | 0.01 | 0.15 | 0.06 | 0.10 | 0.06 | 0.24 |
| Indirect | NA | NA |  | NA |  |  |

Table S4: Direct, total and indirect effects of soil and plant factors on species richness in 2016 in the post-fire site. Both significant and non-significant paths are shown. * = P ≤ 0.10; ** = P ≤ 0.05; *ꜞ = P ≤ 0.005; NA = not applicable

| Effects | Estimates | SE | Std. estimates |
| --- | --- | --- | --- |
| Soil OM → Species Richness |  |  |  |
| Total | -9.21** | 2.29 | -0.54 |
| Direct | -6.90*ꜞ | 2.00 | -0.41 |
| Indirect | -2.31* | 2.36 | -0.13 |
| Soil BD → Species Richness |  |  |  |
| Total | NA | NA | NA |
| Direct | NA | NA | NA |
| Indirect | NA | NA | NA |
| Soil Moisture → Species Richness |  |  |  |
| Total | 0.54** | 0.34 | 0.36 |
| Direct | 0.65*ꜞ | 0.21 | 0.44 |
| Indirect | -0.11 | 0.29 | -0.07 |
| Soil N availability → Species Richness |  |  |  |
| Total | 0.56 | 0.79 | 0.14 |
| Direct | 0.56 | 0.43 | 0.17 |
| Indirect | NA | NA | NA |
| Soil Nutrient availability → Species Richness |  |  |  |
| Total | -11.5** | 6.21 | -0.58 |
| Direct | -14.2*ꜞ | 2.81 | -0.72 |
| Indirect | 2.73 | 2.70 | 0.13 |
| Vegetation cover → Species Richness |  |  |  |
| Total | -0.21 | 0.17 | -0.38 |
| Direct | -0.25*ꜞ | 0.07 | -0.45 |
| Indirect | 0.03* | 0.07 | 0.07 |
| Plant biomass → Species Richness |  |  |  |
| Total | 0.05 | 0.06 | 0.22 |
| Direct | 0.05* | 0.03 | 0.22 |
| Indirect | NA | NA | NA |

**
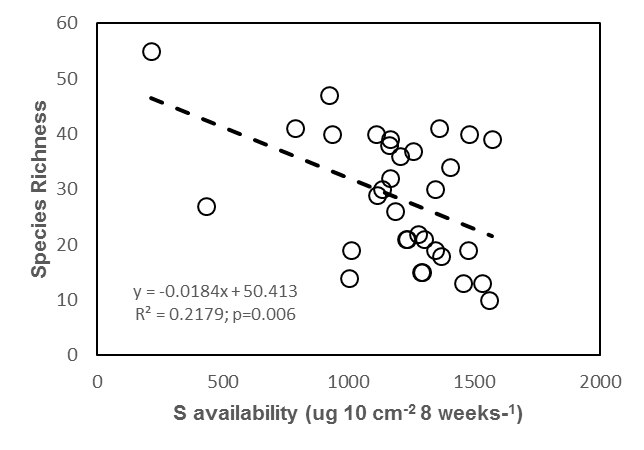

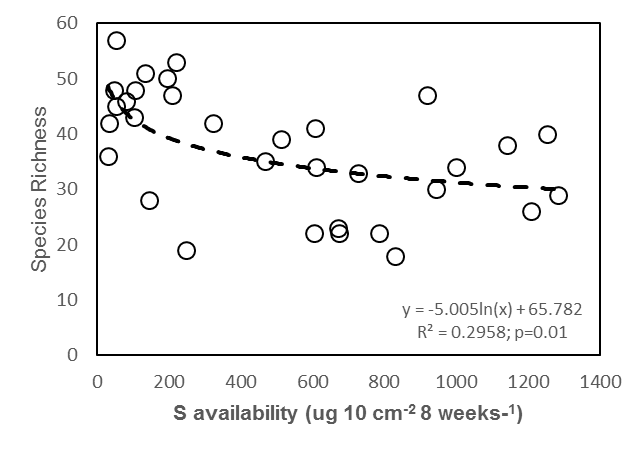

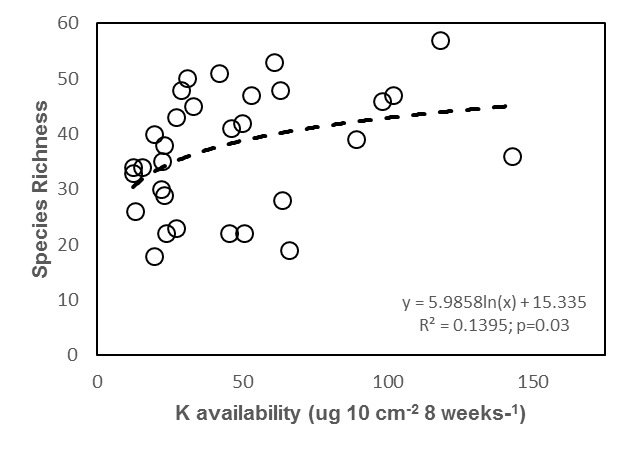

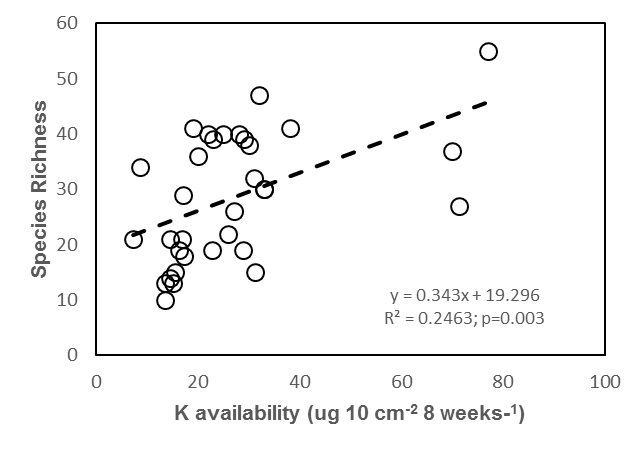

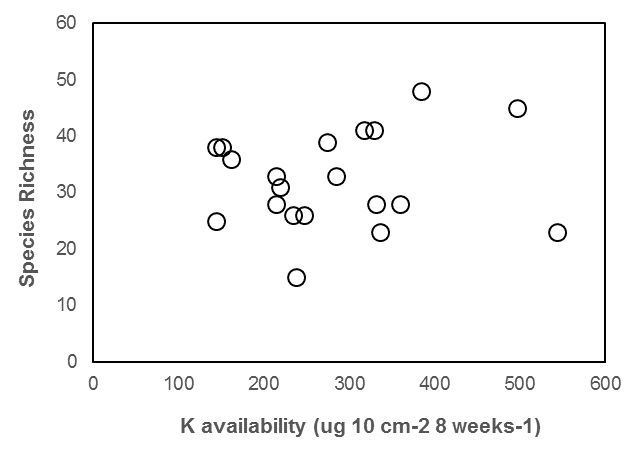
**

FFMM


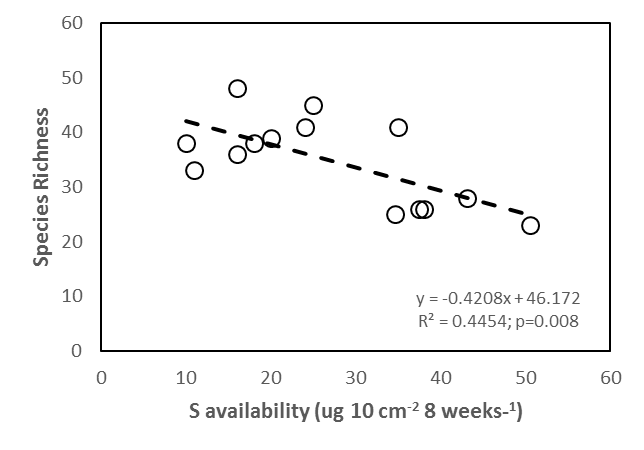


Figure S1: Relationship between species richness and K and S availability in FFMM, PMM and post-fire sites.

Post-fire

PMM


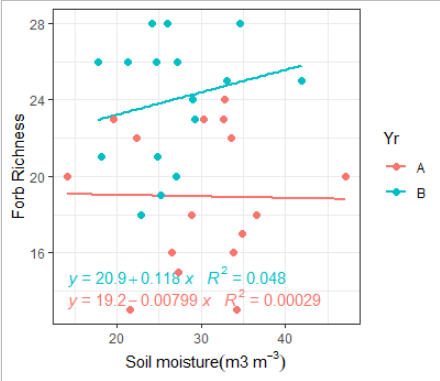

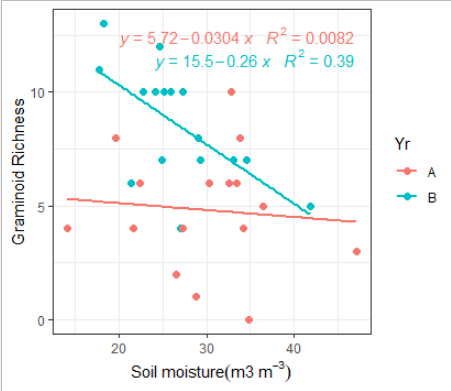

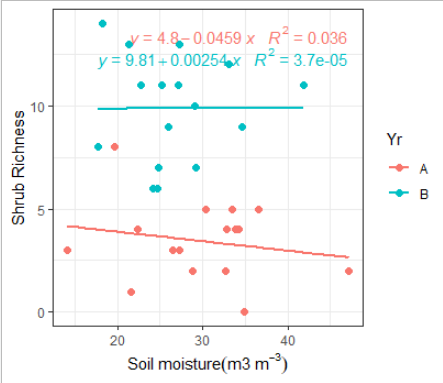

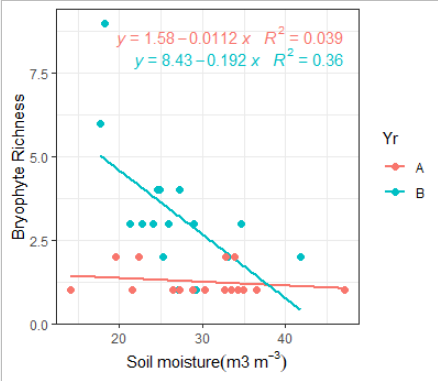


NS

NS

*P<0.05*

NS

*P<0.05*

NS

NS

NS


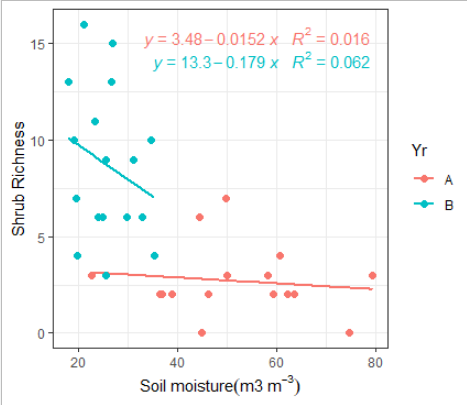

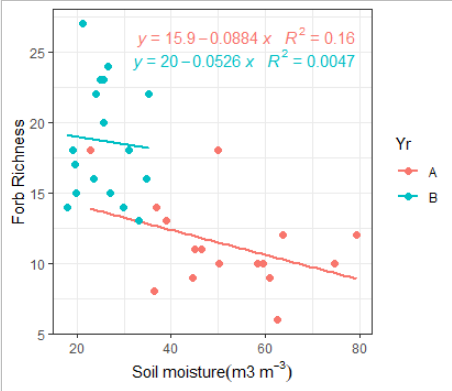

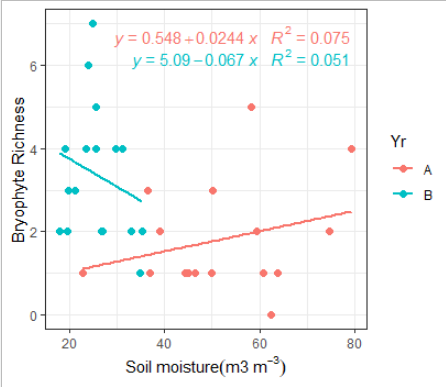

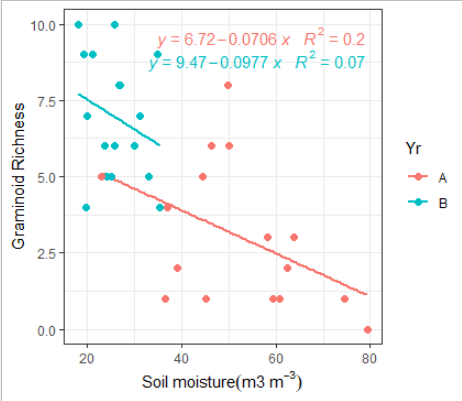


NS

NS

NS

NS

NS; *P=0.11*

*P<0.10*

NS

NS


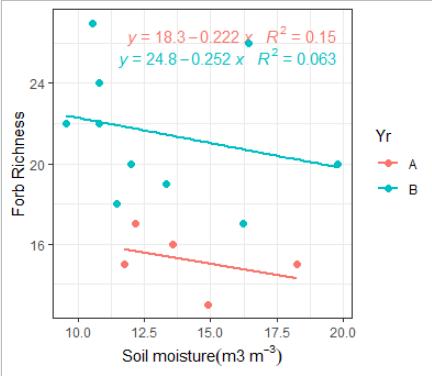

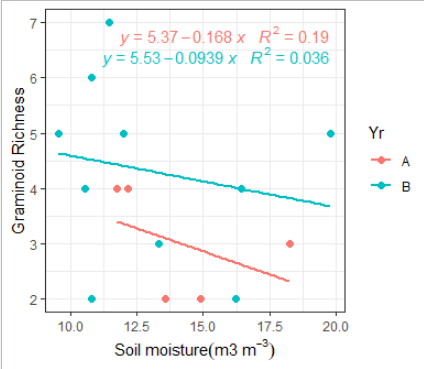

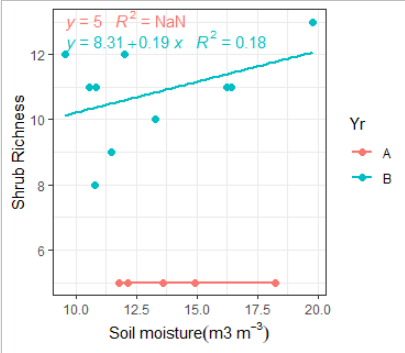

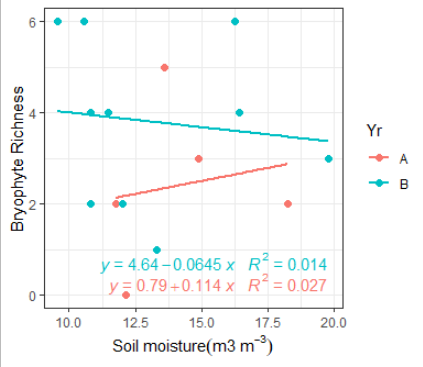


NS

NS

NS

NS

NS

NS

NS

NS

FFMM

PMM

POST-FIRE

Figure S2: Relationship between functional group-level species richness and soil moisture in FFMM, PMM and post-fire sites. Red circle and line= 2013 and Blue circle and line= 2016; NS = Not significant.
